# Combining Diclofenac and Cannabidiol to Enhance the Antibacterial Capacity of Nonantibiotic Drugs Through Potentiation

**DOI:** 10.3390/ijms27135997

**Published:** 2026-07-03

**Authors:** Gratiela Gradisteanu Pircalabioru, Bianca Maria Tihauan, Ciprian Iliescu, Florina Silvia Iliescu

**Affiliations:** 1 eBio-Hub Centre of Excellence in Bioengineering, National University of Science and Technology POLITEHNICA Bucharest, 060042 Bucharest, Romania; gratiela.gradisteanu@icub.unibuc.ro (G.G.P.); bianca.tihauan@upb.ro (B.M.T.); ciprian.iliescu@upb.ro (C.I.); 2 Department of Botany and Microbiology, Faculty of Biology, University of Bucharest, 050095 Bucharest, Romania; 3 Academy of Romanian Scientists, Ilfov 3, 050044 Bucharest, Romania; 4 National Institute for Research and Development in Microtechnologies-IMT Bucharest, 077190 Bucharest, Romania; 5 Faculty of Material Science and Engineering, National University of Science and Technology POLITEHNICA Bucharest, 060042 Bucharest, Romania

**Keywords:** antimicrobial, synergism, potentiation, cannabidiol, diclofenac, personalised medicine

## Abstract

Antimicrobial resistance demands intensive research on new nonantibiotics and drug repurposing to expand the arsenal of antimicrobial agents. The present work analysed the combination of diclofenac (DFNAC) and cannabidiol (CBD) and evaluated its potentiation and its biocompatibility. The formulation’s potency has been tested against *Staphylococcus epidermidis* (*S. epidermidis*), *Staphylococcus aureus* (*S. aureus*), *Escherichia coli* (*E. coli*), and *P. aeruginosa*. The DFNAC-CBD combination showed an evident synergistic effect, a significant decrease in the minimum inhibitory concentration against *Staphylococcus epidermidis*, and an additive effect against *Staphylococcus aureus*, indicating the levels of cooperation between the two compounds. All tested treatments exhibited MBC/MIC ratios ≤ 4, indicating bactericidal activity according to accepted interpretative criteria. Overall, the DFNAC-CBD combination accelerated bacterial killing relative to the individual compounds and exhibited a clear time-dependent antibacterial effect. The combination exhibited no antibacterial activity against Gram-negative strains such as *E. coli* and *Pseudomonas aeruginosa*. 3-(4,5-dimethylthiazol-2-yl)-2,5-diphenyltetrazolium bromide (MTT) and Lactate Dehydrogenase (LDH) assays demonstrated that the antibacterial concentration (3.906 µg/mL) preserves cell viability and membrane integrity. Live/dead staining confirms cell viability and normal morphology. The results indicate that the DFNAC-CBD combination achieves antimicrobial efficacy through bactericidal rather than merely bacteriostatic activities and without inducing significant cytotoxicity. Therefore, the proposed DFNAC-CBD combination has significant potential as a nonantibiotic formula, which with further profile analysis can develop into formulations that can control the use and dosage of common antibiotics.

## 1. Introduction

Rapidly increasing antimicrobial resistance (AMR) is a significant challenge as bacteria evolve mechanisms to evade therapeutic agents, complicating the management of viral, bacterial, parasitic, and fungal diseases, jeopardising public health systems, exacerbating social inequalities, especially in resource-limited areas, impacting the health of animals and plants, and endangering food security [1]. Since AMR-related mortality is projected to rise to over 39 million between 2025 and 2050, the economic implications are enormous, with additional healthcare costs projected at US$1 trillion by 2050 and GDP losses up to US$3.4 trillion annually by 2030 [2,3]. The 2022 Global Antimicrobial Resistance and Use Surveillance System (GLASS) highlighted alarming levels of resistance among bacterial pathogens, including *E. coli* and *Staphylococcus aureus* [4]. Leveraging plants that produce secondary metabolites could provide innovative antibiotics to combat this resistance [5]. Thus, exploring natural products [6] and drug repurposing [7] could support strategies to tackle the pressing threat of multidrug resistance. In this context, some of the FDA-approved nonantibiotic medication classes, including nonsteroidal anti-inflammatory drugs (NSAIDs), antidepressants, antiplatelets, antipsychotics, and statins, have antibacterial effects [8,9] against both Gram-positive and Gram-negative bacteria [10,11,12] and can play essential parts.

Diclofenac sodium (DFNAC), a potent nonsteroidal anti-inflammatory agent, also exhibits antibacterial activity [13] and has various applications [14]. DFNAC alone exhibited activity against drug-sensitive and drug-resistant clinical isolates of *Staphylococcus aureus*, *Listeria monocytogenes*, *Escherichia coli*, and *Mycobacterium* spp. [15]. Also, when combined with streptomycin [15], curcumin [16], gentamicin, or ampicillin [17,18] has potential for the management of problematic antibiotic-resistant Gram-positive and Gram-negative bacterial infections. Furthermore, DFNAC–kanamycin and DFNAC–tetracycline demonstrated additive interactions and promising control of *Escherichia coli* and *Staphylococcus aureus* biofilms [19]. Notably, synergistic effects have been observed between DFNAC and Anidulafungin on tested biofilms [20], DFNAC and Pelargonium graveolens against *Candida parapsilosis* (*C. parapsilosis*) [21], and DFNAC and colistin against multidrug-resistant *Acinetobacter baumannii* (*A. baumannii*) [22]. DFNAC, in combination with *β*-lactams, inhibits methicillin-resistant *Staphylococcus aureus* (MRSA)-associated biofilm formation on implants [23] and can be used in combination with antibiotics as an anti-virulence agent against multidrug-resistant (MDR)-MRSA [24]. However, no potentiation was observed when DFNAC was tested as an adjuvant to antibiotic therapy (cefuroxime, chloramphenicol) against 10 strains of *S. aureus* [25].

Meanwhile, the discovery of phyto-cannabinoids and the identification of their chemical structure, characterised by a lipid structure featuring alkyl-resorcinol and monoterpene moieties, led to the exploration of the endocannabinoid system as the main pharmacological target for cannabinoids [26,27]. Despite ongoing debates about the legal status of *Cannabis*, there is evidence for its useful therapeutic applications in modern medicine, from nausea [28], pain [29,30], Parkinson’s disease and multiple sclerosis-related symptoms [31,32], to sleep disorders [33] and antibacterial and antifilm activities against a broad spectrum of Gram-positive bacteria and some Gram-negative bacteria, including multidrug-resistant (MDR) strains [27,34]. Therefore, distinct *Cannabis*-derived molecules expand the repertoire of antibiotic-independent treatment options [35]. For instance, CBD–polymyxin B exerted an antimicrobial effect against *E. coli* [36], ultrapure CBD–polymyxin B showed potentiated antibacterial activity against *Klebsiella pneumoniae *(*K. pneumoniae*), *Escherichia coli*, and *Acinetobacter baumannii*, including four antibiotic-resistant *K. pneumoniae* isolates [37]. Moreover, CBD–ampicillin and CBD–polymyxin B showed potential synergism against *S. Typhimurium* [38], and CBD–colistin against *A. baumannii* [27,34], with additive and/or synergistic effects against LOS-expressing Gram-negative diplococci; however, no potential synergism between CBD and kanamycin has been found [27,34]. While some studies have recognised CBD as a powerful, low-resistance-inducing antibiotic against Gram-positive bacteria, others have expressed caution due to inconsistencies in bacterial sensitivity and limitations in treating Gram-negative infections [27,34,39]. Since dosage is crucial when therapeutic windows are narrow, potentiation has been considered, and “drug repurposing” [11] leverages synergism with nonantibiotics to extend the spectrum.

The present work explored the combination of DFNAC and CBD and their potential as an alternative antibiotic-independent treatment and prevention strategy in combatting bacterial infections caused by *Staphylococcus epidermidis*, *Staphylococcus aureus*, *Escherichia coli* and *Pseudomonas aeruginosa*. With acceptable biocompatibility and potentiation of antibiotic activity through complementary antimicrobial activity, the combination of DFNAC and CBD shows potential for further studies and targeted applications.

## 2. Results

The proposed formulation combining DFNAC and CBD has been evaluated for its antimicrobial effects against representative Gram-positive and Gram-negative bacteria as well as its biocompatibility.

### 2.1. Antibacterial Activity of DFNAC and CBD Combination

#### 2.1.1. The Antimicrobial Effect Against Gram-Positive Bacteria

**Figure 1:**
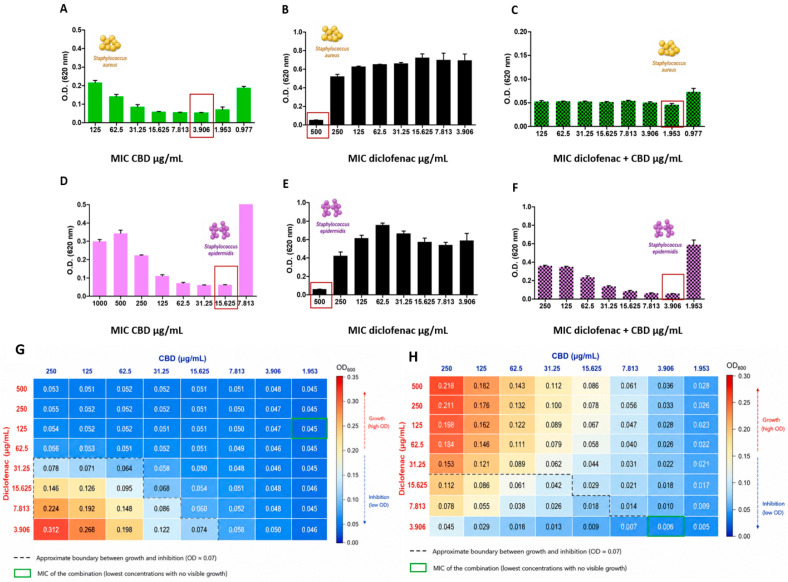
(**A**) MIC of CBD, (**B**) MIC of DFNAC, and (**C**) MIC of the DFNAC-CBD combination. (**D**–**F**) Antibacterial activity against *Staphylococcus epidermidis*. The red boxes indicate the optical densities at concentrations relevant to MIC of CBD, DFNAC and DFNAC-CBD. (**G**) Checkerboard assay illustrating the interaction between DFNAC and CBD against *S. aureus*. The green box indicates the MIC combination (CBD 1.953 µg/mL + DFNAC 125 µg/mL) corresponding to complete inhibition (OD_600_ = 0.045). (**H**) Checkerboard assay for *S. epidermidis*. Optical density (OD_600_) values are represented as heatmaps, where blue indicates bacterial growth inhibition (low OD) and red indicates bacterial proliferation (high OD). The dashed line represents the approximate boundary between growth and inhibition (OD ≈ 0.07). The fractional inhibitory concentration index (FICI) for *S. aureus* was 0.75, indicating an additive interaction.

The antibacterial activity of DFNAC, CBD, and their combination was evaluated against *Staphylococcus aureus* and *Staphylococcus epidermidis* using broth microdilution and checkerboard assays (Figure 1).

For *S. aureus*, CBD alone exhibited antibacterial activity with a MIC of 3.906 µg/mL (Figure 1A), while DFNAC showed inhibitory effects only at high concentrations (MIC = 500 µg/mL; Figure 1B). The DFNAC-CBD combination (Figure 1C) resulted in a modest improvement in antibacterial activity, reflected by reduced optical density values at lower concentrations. The checkerboard analysis (Figure 1G) confirmed an additive interaction, with a FICI value slightly higher than 0.5, indicating partial cooperation between the two compounds without true synergism [40].

In contrast, *S. epidermidis* displayed a different susceptibility profile. CBD alone showed a higher MIC (15.625 µg/mL; Figure 1D), while DFNAC again exhibited weak antibacterial activity (Figure 1E). However, the combination (Figure 1F) significantly enhanced bacterial inhibition, reducing the effective concentration required to achieve complete growth suppression. The checkerboard heatmap (Figure 1H) clearly demonstrates a shift toward lower OD values across combined concentrations, with the MIC of the combination identified at 3.906 µg/mL for both compounds. The calculated FICI value of 0.258 indicates a strong synergistic interaction, corresponding to a 4-fold reduction in the CBD MIC. These results highlight a strain-dependent response to the DFNAC-CBD combination. While *S. aureus* showed only additive effects, *S. epidermidis* exhibited pronounced synergism, suggesting differences in cell envelope structure, membrane permeability, or metabolic sensitivity between the two species.

Overall, the checkboard assay data demonstrate that the DFNAC-CBD combination is particularly effective against *S. epidermidis* (significant decrease in MIC observed in the combination treatment compared with the individual compounds), with synergistic activity (FIC index < 0.5), whereas only additive effects (FIC index > 0.5) are observed against *S. aureus*.

**Figure 2:**
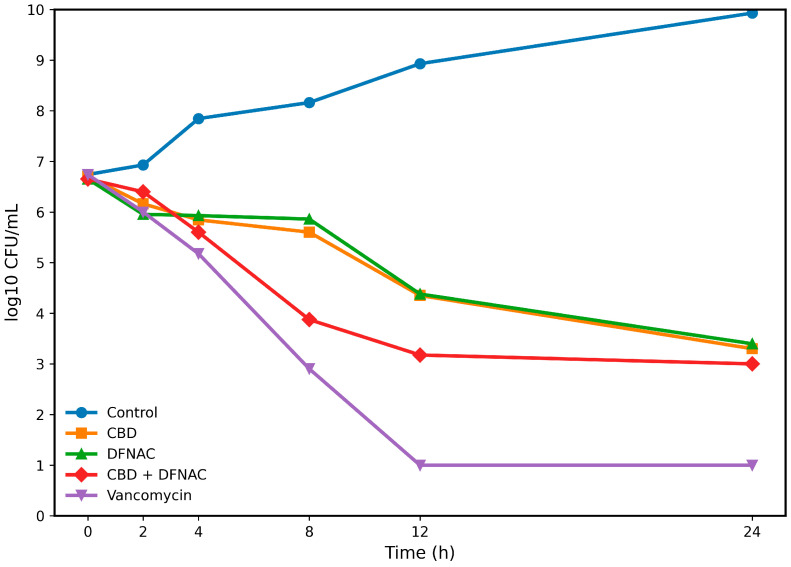
Time-kill kinetics of cannabidiol (CBD), diclofenac sodium (DFNAC), their combination, and vancomycin against *Staphylococcus epidermidis* ATCC 12228.

To further investigate the antibacterial interaction observed in checkerboard assays, time-kill kinetic assays were performed against *Staphylococcus epidermidis* ATCC 12228 using CBD (3.906 µg/mL), DFNAC (500 µg/mL), the DFNAC-CBD combination (3.906 µg/mL CBD + 500 µg/mL DFNAC), and vancomycin (2 µg/mL) as a positive control (Figure 2).

The untreated control exhibited continuous bacterial growth throughout the experiment, increasing from approximately 5 × 10^6^ CFU/mL at inoculation to nearly 9 × 10^9^ CFU/mL after 24 h. CBD and DFNAC alone produced gradual reductions in viable bacterial counts, reaching approximately 2 × 10^3^ and 2.5 × 10^3^ CFU/mL, respectively, after 24 h.

The DFNAC-CBD combination demonstrated a more rapid antibacterial effect than either compound alone. At 8 h, viable bacterial counts were reduced to approximately 7.5 × 10^3^ CFU/mL, compared with approximately 4 × 10^5^ CFU/mL and 7.25 × 10^5^ CFU/mL for CBD and DFNAC, respectively. This enhanced killing effect was maintained throughout the experiment, with the combination reducing bacterial counts to approximately 1 × 10^3^ CFU/mL after 24 h.

Vancomycin displayed the most pronounced bactericidal activity, reducing bacterial counts below the detection limit after 12 h. Overall, the time-kill assay confirmed that the DFNAC-CBD combination accelerates bacterial killing relative to the individual compounds and exhibits a clear time-dependent antibacterial effect.

To assess whether the antibacterial interaction observed in ATCC reference strains was maintained in clinical isolates, checkerboard assays were performed using two clinical isolates of *Staphylococcus aureus* and two clinical isolates of *Staphylococcus epidermidis* (Table S1).

Among the *S. aureus* isolates, CBD MIC values ranged from 7.813 to 31.25 µg/mL, whereas DFNAC MICs were consistently 1000 µg/mL. The addition of DFNAC reduced the effective CBD concentration in isolate SA-C2 from 31.25 to 15.62 µg/mL, while no reduction was observed for isolate SA-C1. Both isolates exhibited FICI values of 1.5, indicating indifferent interactions according to accepted interpretative criteria (Table S1).

For the *S. epidermidis* isolates, CBD MICs were 31.25 µg/mL, whereas DFNAC MICs ranged from 500 to 1000 µg/mL. In isolate SE-C1, the CBD concentration required for growth inhibition decreased from 31.25 to 3.906 µg/mL in the presence of DFNAC, resulting in a FICI value of 1.13, which was classified as indifferent. In isolate SE-C2, the combination reduced the CBD MIC from 31.25 to 7.813 µg/mL and the DFNAC MIC from 1000 to 500 µg/mL, yielding a FICI value of 0.75 and an additive interaction.

Overall, the interaction profile of the DFNAC-CBD combination ranged from indifferent (FICI 1.13–1.50) to additive (FICI 0.75) among the clinical isolates tested. Although the strong synergistic effect observed in the ATCC *S. epidermidis* strain was not consistently reproduced, the reduction in MIC values observed for several isolates suggests that the antibacterial activity of the combination is not restricted exclusively to reference strains and may be influenced by strain-specific susceptibility characteristics.

To evaluate whether the DFNAC-CBD combination proposed as an antimicrobial agent is predominantly bactericidal (actively kills bacteria) or bacteriostatic (stops bacterial replication without killing), the ratio between the minimum bactericidal concentration (MBC) and the minimum inhibitory concentration (MIC) has been determined. To further characterise the antibacterial activity of cannabidiol (CBD), diclofenac (DFNAC), and their combination, minimum bactericidal concentrations (MBCs) were determined and MBC/MIC ratios were calculated for both *Staphylococcus epidermidis* ATCC 12228 and *Staphylococcus aureus* ATCC 25923 (Figures S1 and S2).

For *S. epidermidis* (Figure S1), CBD exhibited a MIC and MBC of 15.625 µg/mL, resulting in an MBC/MIC ratio of 1.0. DFNAC displayed a MIC of 500 µg/mL and an MBC of 2000 µg/mL, corresponding to an MBC/MIC ratio of 4.0. Notably, the DFNAC-CBD combination reduced the MIC to 3.906 µg/mL and exhibited an MBC of 7.813 µg/mL, yielding an MBC/MIC ratio of 2.0. These findings indicate that the combination retained bactericidal activity while requiring substantially lower concentrations than either compound alone.

Similarly, for *S. aureus* ATCC 25923 (Figure S2), CBD demonstrated a MIC of 3.906 µg/mL and an MBC of 7.812 µg/mL, corresponding to an MBC/MIC ratio of 2.0. DFNAC exhibited a MIC of 500 µg/mL and an MBC of 2000 µg/mL, yielding an MBC/MIC ratio of 4.0. The DFNAC-CBD combination maintained antibacterial activity at 3.906 µg/mL CBD in the presence of 500 µg/mL DFNAC and displayed a bactericidal profile with an MBC/MIC ratio ≤ 4.

According to accepted interpretative criteria, all tested treatments exhibited MBC/MIC ratios ≤ 4 and were therefore classified as bactericidal. Importantly, the DFNAC-CBD combination maintained bactericidal activity against both staphylococcal species while reducing the inhibitory concentration required for bacterial growth suppression, supporting the enhanced antibacterial efficacy observed in checkerboard assays. The results highlight the potential of such combinations for selectively targeting Gram-positive pathogens.

#### 2.1.2. The Antimicrobial Effect Against Gram-Negative Bacteria

**Figure 3:**
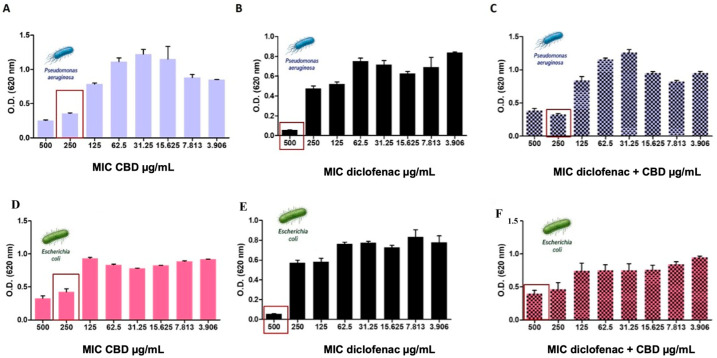
Antibacterial activity of cannabidiol (CBD), diclofenac (DFNAC), and the CBD-DFNAC combination against Gram-negative bacteria, *Escherichia coli* and *Pseudomonas aeruginosa*. (**A**–**C**) Antibacterial activity against *E. coli*: (**A**) MIC of CBD, (**B**) MIC of DFNAC, and (**C**) MIC of the CBD-DFNAC combination. (**D**–**F**) Antibacterial activity against *P. aeruginosa*: (**D**) MIC of CBD, (**E**) MIC of DFNAC, and (**F**) MIC of the CBD-DFNAC combination. Optical density (OD_620_) values were measured following incubation with increasing concentrations of the tested compounds. No minimum inhibitory concentration (MIC) was reached within the tested concentration range for CBD, DFNAC, or the CBD-DFNAC combination against either Gram-negative species. Data are presented as mean ± standard deviation (SD) from three independent experiments. Statistical analysis of OD_620_ values across concentrations was performed using one-way ANOVA followed by Tukey’s multiple-comparison test, with *p* < 0.05 considered statistically significant. The red boxes indicate the optical densities at concentrations relevant to MIC of CBD, DFNAC and DFNAC-CBD.

Both *E. coli* and *P. aeruginosa* exhibited resistance to CBD and the DFNAC-CBD combination, with no MIC reached and no evidence of synergistic interaction (Figure 3). The MIC obtained (Figure 3) under the same conditions show that, indeed, markedly different behaviour was observed for *Pseudomonas aeruginosa* and *E. coli*, in which neither CBD alone nor the DFNAC-CBD combination achieved a minimum inhibitory concentration within the tested range. Consequently, FICI values could not be determined. These results indicate a lack of antibacterial efficacy and absence of synergistic interaction against this strain.

Overall, these findings demonstrate that the effectiveness of the DFNAC-CBD combination is strongly dependent on bacterial species. The observed synergistic effect against *S. epidermidis*, combined with additive activity against *S. aureus* and lack of efficacy against *P. aeruginosa*, underscores the importance of considering bacterial physiology and resistance mechanisms when evaluating combination therapies. This strain-specific response suggests that such combinations may be particularly valuable against susceptible Gram-positive pathogens, while alternative strategies are required to target inherently resistant Gram-negative bacteria.

### 2.2. Biocompatibility of DFNAC-CBD Combination

**Figure 4:**
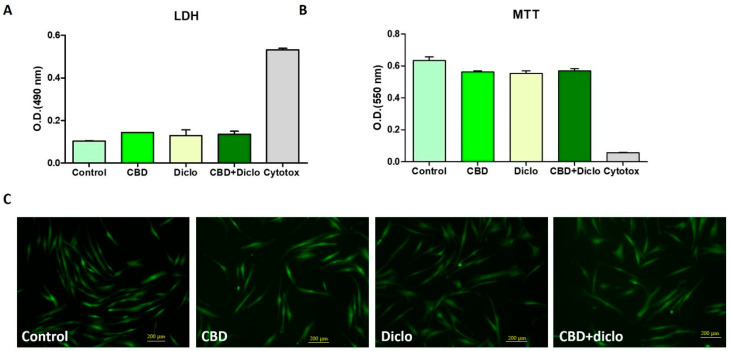
Biocompatibility assessment of CBD, DFNAC, and their combination on human dermal fibroblasts (HDFs). (**A**) Lactate dehydrogenase (LDH) assay evaluating cell membrane integrity. LDH release is expressed as optical density (OD_490_ nm), with higher values indicating increased cytotoxicity. The cytotoxic control represents maximum LDH release. Reference subtraction was performed. (**B**) MTT assay assessing cellular metabolic activity. Cell viability is expressed as optical density (OD_550_ nm), proportional to the number of metabolically active cells. The cytotoxic control shows a marked reduction in viability. (**C**) Live/dead fluorescence imaging of HDF cells after exposure to the tested compounds. Viable cells are stained green (calcein-AM), while dead cells are stained red (ethidium homodimer). Representative images show preserved cell morphology and a predominance of viable cells in all treated groups (>60% at 32 µg/mL, >85% at 3.906 µg/mL), comparable to the control. Overall, the results indicate that CBD, DFNAC, and their combination maintain high cell viability with minimal cytotoxic effects at the tested concentration. Background correction was performed by subtracting the reference absorbance values from the corresponding measurement values.

The cytotoxic effects of the DFNAC-CBD combination (3.906 µg/mL) on HDF cells were further evaluated using the LDH assay, which reflects cell membrane integrity. As shown in Figure 4, all tested samples exhibited low LDH release than the positive control, indicating minimal membrane damage. The DFNAC-CBD combination induced only a slight increase in LDH release relative to the negative control, suggesting limited cytotoxicity. Importantly, the LDH levels remained significantly lower than those observed for the positive control, confirming that the treatment does not cause substantial cell lysis or membrane disruption. 

The overall trend observed in the LDH assay is consistent with the MTT results, supporting the conclusion that the tested concentration maintains high cell viability while inducing only minor cytotoxic effects. The result indicates that the antibacterial MIC does not compromise cell membrane integrity to a biologically significant extent. Taken together, the low LDH release and preserved metabolic activity demonstrate that the DFNAC-CBD combination exhibits good cytocompatibility with HDF cells, reinforcing its potential for biomedical applications that require both antimicrobial efficacy and host cell safety. Quantitative analysis of live/dead staining confirmed the predominance of viable cells in all treatment groups. Cell viability remained above 90% following exposure to CBD, DFNAC, and the CBD-DFNAC combination, with no significant differences compared with untreated controls (Table S2). These observations were consistent with the MTT and LDH assays, further supporting the biocompatibility of the tested compounds.

To further evaluate the safety profile of the tested compounds, the effects of increasing concentrations of CBD, DFNAC, and the CBD-DFNAC combination on HDF cell viability were assessed using the MTT assay (Figure S3). Cells were exposed to concentrations ranging from 3.906 to 32 µg/mL for 24 h.

At the antimicrobial concentration identified in the checkerboard assays (3.906 µg/mL), all treatments maintained high levels of cell viability, exceeding 85% relative to untreated controls. A gradual concentration-dependent decrease in metabolic activity was observed with increasing concentrations of CBD, DFNAC, and the combination. However, the reduction in viability remained moderate across the tested concentration range.

Importantly, even at the highest concentration tested (32 µg/mL), cell viability remained above 60% for all treatment groups, indicating limited cytotoxicity toward HDF cells. As expected, the cytotoxic control (Cytotox) produced a marked reduction in cell viability, confirming the sensitivity of the assay.

Overall, these findings demonstrate that the antibacterial concentrations used in the present study are well tolerated by human dermal fibroblasts and support the favourable cytocompatibility profile of the CBD-DFNAC combination. Furthermore, the maintenance of relatively high cell viability across a concentration range extending beyond the effective antimicrobial dose suggests a reasonable safety margin for future therapeutic applications.

## 3. Discussion

The rise of antibiotic resistance in bacterial pathogens is reducing the effectiveness of essential antibiotics used in invasive surgery and chemotherapy, complicating treatment for normally manageable diseases. To address this, developing new antibacterials and identifying molecules that enhance the efficacy of existing antibiotics are critical priorities.

This study evaluated the antimicrobial responses of various bacterial strains, particularly focusing on the potential synergistic effects of DFNAC and CBD while considering drug repurposing [41] and previous research [42,43]. Some findings suggest that CBD acts as a “potentiator,” improving standard antibiotics’ effectiveness against resistant strains, while others indicate that it may be an effective standalone antibacterial agent for topical use [44]. Other studies highlight DFNAC’s notable antibacterial and anti-inflammatory effects [45]. Our results demonstrated the synergistic interaction against *Staphylococcus epidermidis* when combining DFNAC and CBD, significantly enhancing antibacterial efficacy and substantially reducing the minimum inhibitory concentration (MIC). Conversely, when tested against *Staphylococcus aureus*, the combination exhibited an additive effect, indicating partial cooperation between DFNAC and CBD.

Indeed, research has been directed at identifying antimicrobial candidates to combat the rising antibiotic resistance in Gram-positive bacteria. Given the serious infections caused by *Staphylococcus aureus*, investigating natural products like CBD [36,46,47,48] is a logical step, and further evidence of in vivo efficacy is warranted [49,50]. However, pharmacodynamic studies are required to assess drug–drug interactions for a continuous evaluation of CBD’s benefits [43,44]. For instance, a four-fold reduction in the minimum inhibitory concentration (MIC) shows that only a small amount of CBD is needed to inhibit bacterial growth, highlighting its efficacy, especially in combination therapies or as a structural derivative. This reduction is crucial for reviving susceptibility in antimicrobial-resistant strains, broadening CBD’s therapeutic potential even when used alone. Moreover, a lower active dose reduces cytotoxicity risks and enhances safety. CBD has proven safe for human keratinocytes and erythrocytes at concentrations well above its antibacterial MIC, providing a significant margin of safety. However, achieving effective CBD concentrations in vivo depends on the method of administration. Topical administration is especially effective against *Staphylococcus epidermidis*, often related to skin and surgical infections, as creams can deliver CBD directly to the site, achieving necessary MICs. In contrast, systemic administration for internal infections can be challenging. Oral routes struggle with low bioavailability (about 10–20%) due to first-pass metabolism. To achieve therapeutic concentrations for systemic infections, innovative approaches like intravenous delivery or lipid-based nanocarriers may be necessary to enhance tissue penetration. Although the four-fold reduction in CBD MIC against *S. epidermidis* highlights the potentiating effect of DFNAC, the clinical relevance of this finding must be interpreted in the context of CBD pharmacokinetics. While such concentrations may be challenging to achieve through systemic administration, they are potentially attainable in topical, local, or biomaterial-based delivery systems. Consequently, the observed synergistic interaction may be particularly relevant for localised antimicrobial applications, including wound management, implant coatings, and surface-associated infections. Further pharmacokinetic and in vivo studies are required to determine whether the effective concentrations identified in vitro can be achieved and maintained at sites of infection.

Another question, however, revolves around the rising issue of whether the antibacterial effect stems from bactericidal or bacteriostatic actions. The ratio of minimum bactericidal concentration (MBC) to minimum inhibitory concentration (MIC) is essential for evaluating a formulation’s effectiveness against bacteria. A ratio of 4 or lower, with an MBC close to the MIC [51], indicates a strong bactericidal effect, meaning that the drug not only stops bacterial growth but also kills a majority of the bacteria [52]. These agents typically target critical cellular structures, such as the bacterial cell wall (e.g., penicillin and cephalosporins), or disrupt bacterial DNA, leading to cell death. This makes them crucial for treating severe infections like endocarditis and meningitis, particularly in immunocompromised patients [53]. Conversely, a bacteriostatic mechanism is indicated by a significantly higher MBC compared to the MIC. Here, the drug inhibits growth without killing the bacteria, allowing the immune system to clear the infection. Such agents interrupt reversible processes like protein synthesis (e.g., tetracycline) or folic acid metabolism (e.g., sulfonamides), and are effective for localised infections but less reliable for serious cases [54]. While the MBC/MIC ratio is a vital pharmacological tool, its significance can vary with bacterial strains and testing conditions. Additionally, a drug that is bacteriostatic against one species may be bactericidal against another, underscoring the complexity of these formulations in clinical use. The enhanced efficacy observed in *S. epidermidis* through synergistic interaction between DFNAC and CBD should, now, be interpreted as consistent with rather than proof of complementary actions from DFNAC and CBD leading to enhanced bacterial damage and growth inhibition. Previously published studies proposed CBD-induced alterations of bacterial membrane integrity that primarily disrupt bacterial membrane integrity [55,56], and DFNAC interferences with intracellular metabolic processes [42]. In-depth dedicated membrane permeability assays, together with additional molecular and biophysical approaches, will be required to confirm the mechanism underlying the observed antibacterial interaction between DFNAC and CBD.

Furthermore, previous studies also indicated pairing NSAIDs with antibiotics against *S. epidermidis* and *S. aureus*. DFNAC sodium against *S. epidermidis* indicated a high affinity for *S. epidermidis* proteins and TcaR enzymes and suggested molecular docking [45]. DFNAC combined with gentamicin against *S. aureus* showed a synergistic effect (FIC index 0.38) [15], suggesting efflux pump inhibition. Conversely, ketorolac combined with gentamicin against *S. aureus* biofilms demonstrated synergistic activity against *S. aureus* biofilms (FIC index 0.31), likely through modulation of the biofilm matrix [57].

Despite showing weak-to-moderate direct antibacterial activity (MICs ranging from 32 to 512 µg/mL), based on the checkerboard assays—the primary method for testing synergistic activity—NSAIDs exhibited synergistic effects with various antibiotics like gentamicin, ciprofloxacin, oxacillin, chloramphenicol, cefuroxime, and tetracycline (FIC indices generally < 0.5 to 0.625). Efflux pump inhibition is frequently proposed as an MOA [41]. Moving forward, more molecular studies are essential to validate these findings and explore the potential of NSAIDs as modulators or antibacterial adjuvants. However, challenges remain, such as achieving effective concentrations without toxicity and confirming mechanisms to ensure pharmacokinetic and pharmacodynamic compatibility and specificity and avoid off-target effects. Furthermore, our exploratory evidence indicates that the combination of DFNAC and CBD may become a significant antimicrobial pairing, aligning with the ongoing efforts to refine applications [58,59,60] and establish a regulatory framework [61] for future pharmacological products. It also aims to address methodological inconsistencies in standardised testing methods, culture media, and CBD sources.

Notably, we found no antibacterial activity against the Gram-negative strains *Escherichia coli* and *Pseudomonas aeruginosa*, which reflects their intrinsic resistance and highlights the combination’s inability to overcome the permeability barrier of their outer membranes. The intrinsic resistance of *P. aeruginosa* is well documented. It can be attributed to its highly impermeable outer membrane, efficient efflux pump systems, and adaptive and acquired resistance mechanisms [62], which collectively limit the penetration and activity of many antimicrobial compounds.

While it is widely accepted that CBD is highly effective against Gram-positive bacteria, including MRSA, there is a debate about its efficacy against Gram-negative bacteria. Many studies support the view that CBD is inactive against Gram-negative bacteria due to their protective outer membrane. However, a conflicting hypothesis suggests that certain Gram-negative bacteria—specifically *Neisseria gonorrhoeae*, *Neisseria meningitidis*, and *Legionella pneumophila*—are highly sensitive to CBD. This observation raises the question of whether CBD is a niche Gram-positive agent or a broad-spectrum candidate with tailored applications [27,34,55,63]. In the meantime, dose–response studies against *E. coli* and *S. aureus* indicated that DFNAC reduced metabolic activity without removing biofilm mass at therapeutic concentrations, resulting in significant reductions in colony-forming units within the biofilm as confirmed by Scanning Electron Microscopy [64]. However, the bactericidal effect against Gram-negative and Gram-positive bacteria—evident through inhibition of biofilm formation or reduction in metabolic viability—is limited at clinically relevant concentrations with standardised MBIC or MBEC values missing. The minimum bactericidal concentrations (MBCs) were consistently above 2000 µg/mL, indicating limited bactericidal potential [41]. Therefore, direct and consistent data on membrane disruption as well as in vivo or toxicity study results are still needed for conclusive mapping. To aid in drug formulation profiling, our study provided information on drug–drug interactions and confirmed the biocompatibility of the proposed combination of DFNAC and CBD. We highlighted the biocompatibility profile of the combination on human dermal fibroblasts (HDFs). Both MTT and LDH assays demonstrated that the antibacterial concentration (3.906 µg/mL) preserves cell viability and membrane integrity. Live/dead staining confirmed that most cells remained viable and exhibited normal morphology. The overall results indicate that the combination operates within a therapeutic window, achieving antimicrobial efficacy without inducing significant cytotoxicity. Moreover, the combination shows high biocompatibility. Investigations reported that CBD decreased the overall acidity levels in rats treated with DFNAC, suggesting that cannabidiol effectively treated the condition [65], or provided hepatoprotection against DFNAC sodium-induced hepatotoxicity [66]. However, the increasing interest in cannabis also requires a deeper understanding of the potential interactions between CBD and NSAIDs based on current knowledge and evidence [67,68].

Therefore, further rigorous quantitative biofilm assays, mechanistic membrane studies, and validation through clinical or animal models are essential before NSAIDs [42] and CBD [69] can be considered viable anti-biofilm adjuvants. Although DFNAC was tested in clinical trials involving patients with uncomplicated urinary tract infections and cellulitis [44], additional studies are necessary to complete its antimicrobial profile. Recent developments in plant-derived and synthetic cannabinoids [59] and NSAIDs [70] support the hypothesis that new therapeutic formulations [71,72] can be designed [73] specifically to address the antimicrobial crisis while meeting clinical applications [74] and regulatory requirements. Moreover, addressing antibiotic resistance through drug reprofiling will enhance formulations for personalised medicine.

Several limitations of the present study should be acknowledged. First, although the antibacterial activity of cannabidiol (CBD), diclofenac sodium (DFNAC), and their combination was evaluated against both reference ATCC strains and a limited number of clinical isolates, the number of clinical strains tested remains relatively small. While the observed antibacterial activity across both reference and clinical isolates supports the potential of the CBD-DFNAC combination, the variability observed among clinical isolates indicates that strain-specific susceptibility profiles may influence the magnitude of the interaction. Furthermore, while the observed antimicrobial and bactericidal effects across both reference and clinical isolates are encouraging, they may not fully capture the phenotypic and genotypic diversity encountered in clinical settings. Therefore, additional studies involving a larger collection of clinical isolates, including multidrug-resistant strains such as MRSA and resistant *Staphylococcus epidermidis* isolates, are required to validate the robustness, spectrum of activity, and translational potential of the CBD–DFNAC combination. Consequently, the present findings should be considered proof-of-concept evidence supporting further investigation toward clinical application.

Second, although checkerboard, MBC/MIC, and time-kill kinetic assays demonstrated enhanced antibacterial activity of the combination, the molecular mechanisms underlying this interaction were not directly investigated. The proposed mechanisms, including CBD-mediated membrane perturbation and DFNAC-associated interference with bacterial metabolic processes, are based on the previous literature and should therefore be considered hypotheses rather than experimentally confirmed mechanisms in the context of the present study. Additional investigations employing membrane permeability assays (e.g., SYTOX Green uptake or propidium iodide staining), transcriptomic analyses, proteomics, and metabolomics will be necessary to elucidate the molecular basis of the observed antibacterial effects.

Third, the study did not include experiments designed to evaluate the emergence of antimicrobial resistance during prolonged exposure to CBD, DFNAC, or their combination. Consequently, the potential of the combination to delay or prevent resistance development compared with monotherapy remains speculative and requires dedicated evolutionary and serial-passage studies.

Another limitation is the lack of activity observed against the Gram-negative strains *Escherichia coli* and *Pseudomonas aeruginosa*. While this finding is consistent with previous reports describing the intrinsic resistance of Gram-negative bacteria to cannabinoids due to the outer-membrane permeability barrier, the present study did not investigate whether membrane-permeabilising agents could restore susceptibility. Future studies should evaluate the DFNAC-CBD combination together with agents such as polymyxin B or colistin to determine whether the antibacterial spectrum can be expanded toward Gram-negative pathogens. In addition, a dedicated Gram-negative antibiotic control was not included in the original experimental design, limiting direct benchmarking of antibacterial efficacy against established therapies.

An additional limitation of the present study concerns the concentration range evaluated in the cytocompatibility assays. Cell viability was assessed using concentrations encompassing the antibacterial MIC and extending up to 32 µg/mL, which represents approximately an eight-fold increase over the effective antimicrobial concentration. While this range was selected to evaluate the safety margin associated with biologically relevant antibacterial doses, it does not establish the complete toxicological profile of the formulation. Future investigations should include substantially higher concentrations, prolonged exposure periods, additional primary human cell types, and in vivo biocompatibility studies to define the maximum tolerated dose and therapeutic window more comprehensively.

Finally, the current findings are based exclusively on in vitro experiments. The pharmacokinetic behaviour, tissue distribution, stability, and in vivo antibacterial efficacy of the DFNAC CBD combination remain unknown. Although the concentrations identified in vitro may be difficult to achieve through systemic administration, they may be attainable in localised applications such as wound dressings, biomaterial coatings, implant-associated infections, and topical formulations. Therefore, future studies should focus on formulation development, pharmacokinetic characterisation, and validation in relevant animal and non-animal models before clinical translation can be considered.

Taken together, the present findings provide proof-of-concept evidence supporting the antibacterial potential of the DFNAC-CBD combination against Gram-positive staphylococci. The pronounced synergistic interaction observed against *S. epidermidis*, compared with the additive effect detected in *S. aureus*, may reflect intrinsic biological differences between these species. Although both are Gram-positive staphylococci, they differ in membrane composition, regulatory pathways, biofilm-associated proteins, and stress-response mechanisms. CBD is believed to primarily affect membrane integrity, whereas diclofenac may interfere with intracellular metabolic processes and bacterial regulatory networks. The greater susceptibility of *S. epidermidis* to the combined action of these compounds may explain the stronger reduction in MIC observed in this species. In contrast, the more complex adaptive responses of *S. aureus* may attenuate the potentiating effect of the combination, resulting in an additive rather than synergistic interaction. Further mechanistic investigations will be necessary to clarify the molecular basis of these species-specific responses. Also, additional pharmacological and translational studies are required to fully establish complex clinical perspectives.

## 4. Materials and Methods

### 4.1. Antibacterial Activity of DFNAC and CBD Combination

The antimicrobial interaction between diclofenac sodium (DFNAC) (certified reference material, CAS 15307-79-6, Merck, Sigma-Aldrich, Bucharest, Romania, dissolved in PBS) and cannabidiol (CBD) (99.7% purity, Happease, Cluj Napoca, Romania, dissolved in DMSO) was evaluated against ATCC bacterial strains cultured in tryptic soy broth (TSB). Bacterial suspensions were prepared from overnight cultures and adjusted to the required inoculum density. Serial two-fold dilutions of DFNAC, CBD, and DFNAC-CBD combinations were prepared in TSB in sterile 96-well microplates. Wells containing bacteria without treatment served as growth controls, while wells containing TSB and tested compounds without bacteria were used as blanks. Plates were incubated at 37 °C for 24 h, after which bacterial growth was evaluated by measuring optical density at 620 nm. The minimum inhibitory concentration (MIC) was defined as the lowest concentration showing no visible bacterial growth and OD values comparable to the blank control. The interaction between DFNAC and CBD was assessed using the fractional inhibitory concentration index (FICI), calculated as follows:

FICI = (MIC CBD in combination/MIC CBD alone) + (MIC DFNAC in combination/MIC DFNAC alone)(1)

The interaction was interpreted as synergistic when FICI ≤ 0.5, additive when 0.5 < FICI ≤ 1, indifferent when 1 < FICI ≤ 4, and antagonistic when FICI > 4.

A positive control to all checkerboard and MIC experiments has been added; 2 μg/mL vancomycin has been used as a standard antibiotic positive control for *S. aureus* to benchmark the relative potency of the DFNAC-CBD combination against established antimicrobials.

Briefly, overnight bacterial cultures were grown in tryptic soy broth (TSB) at 37 °C and adjusted to approximately 1 × 10^6^ CFU/mL. Aliquots of the bacterial suspension were exposed to CBD (3.906 µg/mL), DFNAC (500 µg/mL), the DFNAC-CBD combination (3.906 µg/mL CBD + 500 µg/mL DFNAC), or vancomycin (2 µg/mL), which served as a positive antibacterial control. Untreated bacterial cultures were included as growth controls. Cultures were incubated at 37 °C under aerobic conditions with gentle agitation. Samples were collected at 0, 2, 4, 8, 12, and 24 h following treatment. At each time point, serial ten-fold dilutions were prepared in sterile phosphate-buffered saline (PBS), and 100 µL aliquots were spread onto Mueller–Hinton agar plates. Plates were incubated at 37 °C for 18–24 h, after which colonies were counted and expressed as colony-forming units per millilitre (CFU/mL). Results were converted to log_10_ CFU/mL for graphical representation and comparison of bacterial killing kinetics. The time-kill assay was conducted at the concentration corresponding to the minimum inhibitory concentration (MIC) identified in the checkerboard experiments, allowing assessment of the temporal antibacterial activity of the CBD-DFNAC combination under biologically relevant conditions.

### 4.2. Biocompatibility of DFNAC-CBD Combination

Cell viability and metabolic activity were assessed using the MTT Cell Proliferation Assay Kit (Roche Diagnostics) according to the manufacturer’s protocol. Human dermal fibroblasts (HDFs) were obtained from ATCC (ethical approval was obtained). HDFs were seeded in 24-well plates at a density of 1 × 10^4^ cells/well and cultured in Dulbecco’s Modified Eagle Medium (DMEM) supplemented with 10% fetal bovine serum (FBS) under standard conditions (37 °C, 5% CO_2_, humidified atmosphere). After 24 h to allow cell attachment, the culture medium was replaced, and the cells were exposed to the tested DFNAC-CBD mixtures at the selected concentrations. Following 24 h incubation, the culture medium was removed, and the cells were gently washed with phosphate-buffered saline (PBS). MTT reagent was added to each well at a final concentration of 0.5 mg/mL and incubated for 4 h at 37 °C to permit the formation of formazan crystals by metabolically active cells. Subsequently, the crystals were dissolved using the supplied solubilisation solution, and absorbance was measured at 550 nm using a Multiskan FC microplate reader (Thermo Fisher Scientific, Bucharest, Romania). The obtained absorbance values were considered directly proportional to the number of viable cells and overall metabolic activity.

Cytotoxicity and membrane integrity were further evaluated using the Lactate Dehydrogenase (LDH) Cytotoxicity Detection Kit (Roche Diagnostics, Mannheim, Germany), according to the manufacturer’s instructions. The LDH assay quantifies the release of lactate dehydrogenase into the culture medium following damage to the plasma membrane and is widely used as an indicator of cell membrane integrity and cytotoxicity. HDF cells were seeded in 96-well plates and exposed to CBD, DFNAC, or CBD-DFNAC formulations for 24 h under standard culture conditions (37 °C, 5% CO_2_). Following incubation, 50 µL of culture supernatant from each well was carefully transferred to a fresh 96-well microplate. Subsequently, 100 µL of freshly prepared reaction mixture was added to each sample and incubated for 15–20 min at room temperature in the dark. The enzymatic reaction was stopped according to the manufacturer’s protocol, and absorbance was measured at 490 nm with a reference wavelength of 620 nm using a microplate reader. Background correction was performed by subtracting the reference absorbance values from the corresponding measurement values. Untreated cells served as the negative control representing spontaneous LDH release, while cells treated with Triton X-100 (0.01%, *v*/*v*) (Sigma Aldrich, Bucharest, Romania) for 15 min before analysis served as the positive cytotoxicity control (CytoTox® (Promega Corporation, Dexter Com S.R.L., Bucharest, Romania)). Triton X-100 is a non-ionic detergent that disrupts cellular membranes, resulting in complete cell lysis and maximal LDH release. This control was used to define 100% cytotoxicity and to validate the sensitivity of the assay.

Cell viability and morphology were additionally analysed using the Live/Dead Viability/Cytotoxicity Kit (Invitrogen, Thermo Fisher Scientific). HDF cells were seeded at a density of 1 × 10^4^ cells/well and incubated with the CBD–diclofenac mixtures for 24–72 h under standard culture conditions (37 °C, 5% CO_2_). After treatment, the culture medium was removed and the cells were carefully washed with PBS. A staining solution containing calcein-AM, which labels viable cells with green fluorescence, and ethidium homodimer-1, which stains dead cells red, was prepared according to the manufacturer’s recommendations and added to each well. Samples were incubated for 30–60 min in the dark at room temperature prior to imaging. Fluorescence images were acquired using a Zeiss Axioscope fluorescence microscope (Carl Zeiss Instruments SRL, Bucharest, Romania)) equipped with an AxioCam imaging system (Carl Zeiss Instruments SRL, Bucharest, Romania)) to evaluate cell viability, morphology, and cellular attachment following exposure to the tested formulation. The quantification of life/dead cell ratios was performed to show the predominance of viable cells in all treated groups, compared with the control. HDF cells were exposed to increasing concentrations of CBD, diclofenac, or the CBD–diclofenac combination (3.906, 7.81, 15.625, and 32 µg/mL).

### 4.3. Statistical Analysis

All experiments were performed independently at least two times, and data are presented as mean ± standard deviation (SD). For cell-based assays (MTT, LDH, and live/dead quantification), statistical analyses were performed using GraphPad Prism version 9.4 (GraphPad Software, San Diego, CA, USA). Differences among groups were evaluated using one-way analysis of variance (ANOVA) followed by Tukey’s multiple-comparison test. A *p*-value < 0.05 was considered statistically significant.

Minimum inhibitory concentration (MIC), minimum bactericidal concentration (MBC), and checkerboard assays were interpreted according to Clinical and Laboratory Standards Institute (CLSI) guidelines. Because MIC and MBC determinations represent categorical antimicrobial endpoints rather than continuous quantitative dose–response variables, these data were not subjected to inferential statistical testing. MIC and MBC values are reported as the modal value obtained from three independent experiments or as mean ± SD where appropriate. Synergistic interactions were evaluated using the fractional inhibitory concentration index (FICI).

## 5. Conclusions

Combining DFNAC, a common potent nonsteroidal anti-inflammatory drug, with CBD offers a promising approach to enhance antimicrobial capacity against various bacterial strains. The combination’s combined bactericidal and bacteriostatic actions facilitated a synergistic effect against *S. epidermidis* and an additive effect against *S. aureus*. The lack of antibacterial activity detected against the Gram-negative strains *Escherichia coli* and *Pseudomonas aeruginosa* reflects the need for new complementary MOAs that can simultaneously disrupt bacterial defence mechanisms and prevent the development of resistance. Using a combination of DFNAC and CBD allows for lower, less toxic doses of both substances. However, more data is needed to analyse how co-treatment could reduce the development of resistance compared to single-agent treatment. Moreover, the proposed combination provides direct bacteria killing, helping reduce tissue damage during infection and creating opportunities for personalised, targeted therapeutic approaches. DFNAC and CBD are excellent candidates for repurposing as adjuncts to enhance the efficacy of conventional antibiotics. Further evaluation will enhance the presented profile to validate the combination’s spectrum and expand the clinical applications.

## Data Availability

The datasets generated and/or analysed during the current study are publicly available in the Mendeley Data repository: Gradisteanu G. “CBD-DCFN testing”, Mendeley Data, V1, 2026. https://doi.org/10.17632/6hv6tzgjg3.1.

## Supplementary Materials

The following supporting information can be downloaded at https://www.mdpi.com/article/10.3390/ijms27135997/s1.

## Abbreviations

The following abbreviations are used in this manuscript:

AMR	antimicrobial resistance
CBD	cannabidiol
DFNAC	diclofenac
FICI	fractional inhibitory concentration index
HDF	human dermal fibroblast
LDH	lactate dehydrogenase
MIC	minimum inhibitory concentration
MBC	minimum bactericidal concentrations
MOA	mechanism of action
NSAID	nonsteroidal anti-inflammatory drug
OD	optical density
TSB	tryptic soy broth
