# Supplementary material for: Combining Diclofenac and Cannabidiol to Enhance the Antibacterial Capacity of Nonantibiotic Drugs Through Potentiation
Source: Int J Mol Sci. 2026 Jul 3;27(13):5997. doi: 10.3390/ijms27135997 (PMC13361921; doi:10.3390/ijms27135997)
Supplement: Supplementary file 1 [file ijms-27-05997-s001.zip › ijms-4366319-supplementary.pdf]

Supplementary

Species	Isolate ID	CBD MIC (µg/mL)	DFNAC MIC (µg/mL)	CBD MIC in combination (µg/mL)	DFNAC MIC in combination (µg/mL)	FICI	Interpretation
S. aureus	SA-C1	7.813	1000	7.813	500	1.5	Indifferent
S. aureus	SA-C2	31.25	1000	15.62	1000	1.5	Indifferent
S. epidermidis	SE-C1	31.25	500	3.906	500	1.13	Indifferent
S. epidermidis	SE-C2	31.25	1000	7.813	500	0.75	Additive

Table S1. Minimum inhibitory concentrations (MICs), fractional inhibitory concentration indices (FICI), and interaction profiles of cannabidiol (CBD) and diclofenac (DFNAC) against clinical isolates of *Staphylococcus aureus* and *Staphylococcus epidermidis*.

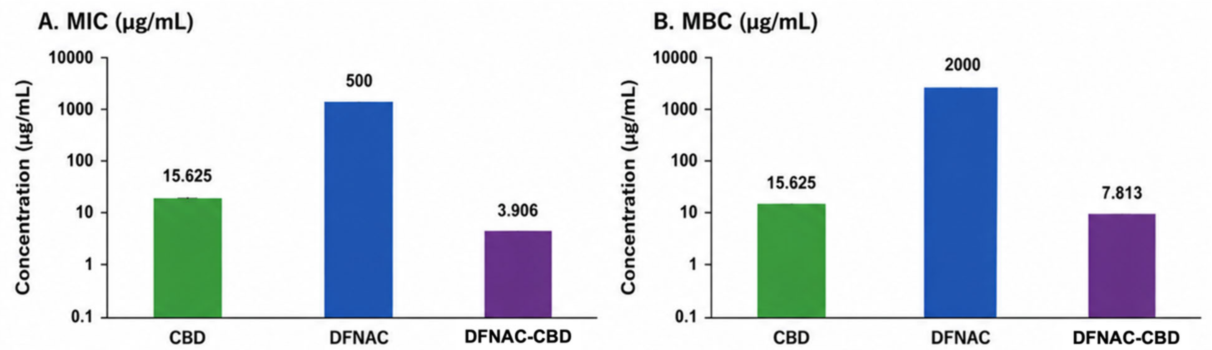

C.

Treatment	MIC (µg/mL)	MBC (µg/mL)	MBC/MIC ratio	Interpretation*
CBD	15.625	15.625	**1.0**	Bactericidal
DFNAC	500	2000	**4.0**	Bactericidal
DFNAC-CBD combination	3.906	7.813	**2.0**	Bactericidal

Figure S1. Minimum inhibitory concentration (MIC), minimum bactericidal concentration (MBC), and MBC/MIC ratios of cannabidiol (CBD), diclofenac (DFNAC), and their combination against *Staphylococcus epidermidis* ATCC strain.

(A) Minimum inhibitory concentrations (MICs) of CBD, DFNAC, and the DFNAC–CBD combination determined by broth microdilution. CBD exhibited a MIC of 15.625 µg/mL, while DFNAC showed antibacterial activity only at higher concentrations (MIC ≈ 500 µg/mL). The combination reduced the effective inhibitory concentration to 3.906 µg/mL.

(B) Minimum bactericidal concentrations (MBCs) determined by subculturing aliquots from growth-inhibited wells onto Mueller–Hinton agar plates. The MBC values were 15.625 µg/mL for CBD, 2000 µg/mL for DFNAC, and 7.813 µg/mL for the DFNAC–CBD combination.

(C) Summary table showing MIC, MBC, and MBC/MIC ratios. According to the accepted interpretation criteria (MBC/MIC ≤ 4), all treatments demonstrated bactericidal activity against S. epidermidis. The DFNAC–CBD combination exhibited the lowest MBC value and maintained a bactericidal profile (MBC/MIC = 2), supporting the enhanced antibacterial efficacy observed in the checkerboard assay.

MIC and MBC values represent the modal concentration obtained from 2 independent experiments. MBC/MIC ratios were interpreted as follows: ≤ 4, bactericidal activity; > 4, bacteriostatic activity.

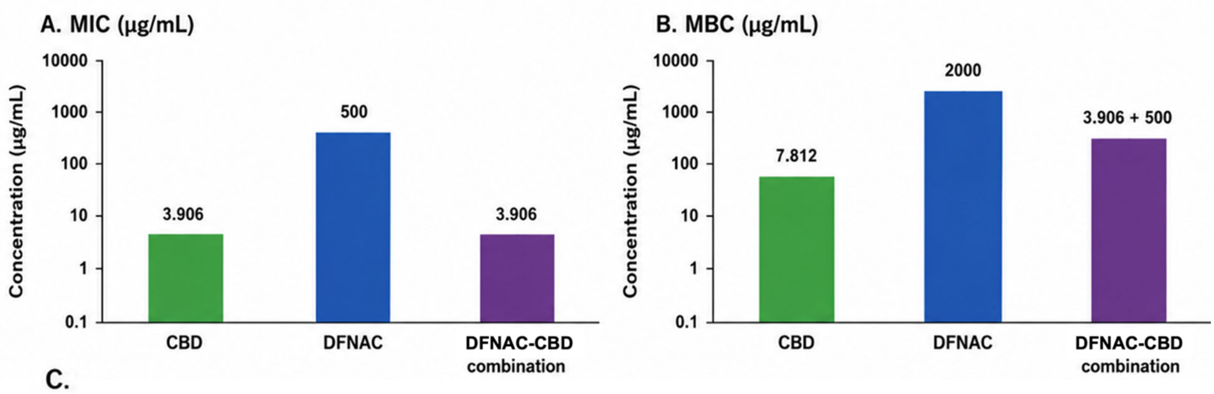

C.

Treatment	MIC (µg/mL)	MBC (µg/mL)	MBC/MIC ratio	Interpretation*
CBD	3.906	7.812	**2.0**	Bactericidal
DFNAC	500	2000	**4.0**	Bactericidal
DFNAC-CBD combination	3.906	3.906 + 500	≤ **4.0**	Bactericidal

Figure S2. Minimum inhibitory concentration (MIC), minimum bactericidal concentration (MBC), and MBC/MIC ratios of cannabidiol (CBD), diclofenac (DFNAC), and their combination against *Staphylococcus aureus* ATCC 25923.

(A) Minimum inhibitory concentrations (MICs) of CBD, DFNAC, and the DFNAC–CBD combination determined by broth microdilution. CBD exhibited antibacterial activity at 3.906 μg/mL, whereas DFNAC inhibited bacterial growth at 500 μg/mL. The DFNAC–CBD combination maintained growth inhibition at 3.906 μg/mL CBD in the presence of 500 μg/mL DFNAC.

(B) Minimum bactericidal concentrations (MBCs) determined by subculturing aliquots from growth-inhibited wells onto Mueller–Hinton agar plates. The MBC values were 7.812 μg/mL for CBD and 2000 μg/mL for DFNAC. For the DFNAC–CBD combination, bactericidal activity was observed at 3.906 μg/mL CBD combined with 500 μg/mL DFNAC.

(C) Summary table presenting MIC, MBC, and MBC/MIC ratios for each treatment. CBD displayed an MBC/MIC ratio of 2.0, while DFNAC exhibited a ratio of 4.0. The DFNAC–CBD combination maintained a bactericidal profile with an MBC/MIC ratio ≤ 4. According to accepted interpretative criteria (MBC/MIC ≤ 4), all treatments demonstrated bactericidal activity against *S. aureus* ATCC 25923. These findings indicate that the antibacterial activity of CBD and the DFNAC–CBD combination is associated with bacterial killing rather than growth inhibition alone.

MIC and MBC values were determined by broth microdilution followed by subculture on Mueller–Hinton agar. Bactericidal activity was defined as an MBC/MIC ratio ≤ 4, whereas ratios > 4 were considered bacteriostatic.

Treatment	Live cells (%)
Control	98.1 ± 1.2
CBD	95.6 ± 2.1
DFNAC	94.8 ± 2.5
CBD+DFNAC	95.2 ± 1.8

Table S2. Live cell quantification using the Image J software

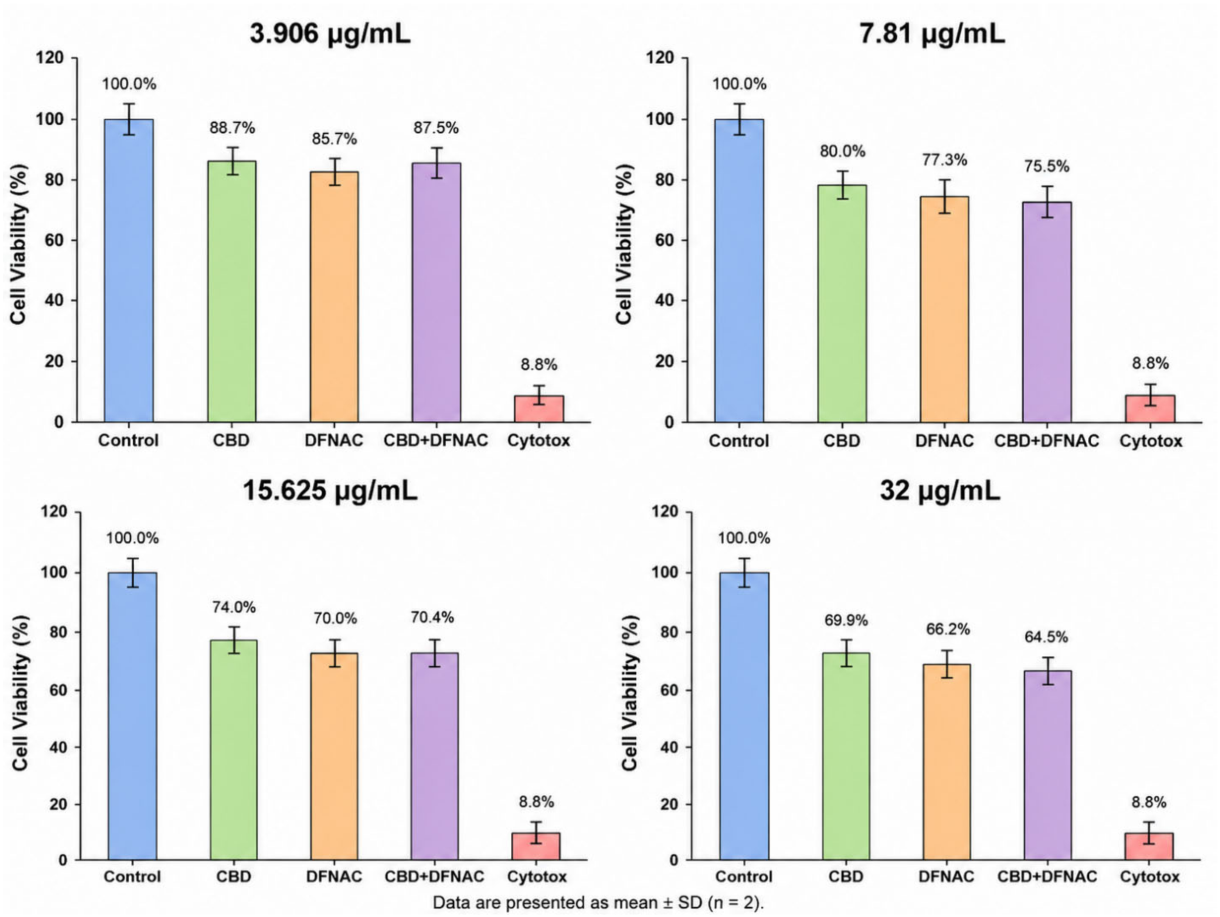

Figure S3. Concentration-dependent effects of cannabidiol (CBD), diclofenac (DFNAC), and their combination (CBD+DFNAC) on human dermal fibroblast (HDF) viability determined by MTT assay. HDF cells were exposed for 24 h to increasing concentrations of CBD, diclofenac, or the CBD–diclofenac combination (3.906, 7.81, 15.625, and 32 µg/mL). Cell viability was assessed by measuring the conversion of MTT to formazan and expressed as percentage viability relative to untreated control cells (set at 100%). The cytotoxic control (Cytotox) served as a positive control for cell death. All treatments maintained relatively high viability at the antimicrobial concentration of 3.906 µg/mL (>85%), while a concentration-dependent reduction in metabolic activity was observed at higher concentrations. Nevertheless, cell viability remained above 60% for all tested compounds and combinations up to 32 µg/mL, indicating acceptable cytocompatibility. Data are presented as mean ± SD from two independent experiments (n = 2).
